# Correlates of property crime in a cohort of recently released prisoners with a history of injecting drug use

**DOI:** 10.1186/s12954-015-0057-y

**Published:** 2015-08-04

**Authors:** Amy Kirwan, Brendan Quinn, Rebecca Winter, Stuart A. Kinner, Paul Dietze, Mark Stoové

**Affiliations:** Centre for Population Health, Burnet Institute, 85 Commercial Rd, Melbourne, Victoria 3004 Australia; School of Public Health and Preventive Medicine, Monash University, Melbourne, Victoria Australia; Melbourne School of Population and Global Health, University of Melbourne, 207 Bouverie Street, Carlton, Melbourne, Victoria 3010 Australia; School of Medicine, University of Queensland, Brisbane, Queensland Australia

**Keywords:** Prison, Injecting, Drug use, Crime

## Abstract

**Background:**

Injecting drug use (IDU) is a strong predictor of recidivism and re-incarceration in ex-prisoners. Although the links between drug use and crime are well documented, studies examining post-release criminal activity and re-incarceration risk among ex-prisoners with a history of IDU are limited. We aimed to explore factors associated with property crime among people with a history of IDU recently released from prison.

**Method:**

Individuals with a history of IDU released from prison within the past month were recruited via targeted and snowball sampling methods from street drug markets and services for people who inject drugs (PWID) into a 6-month cohort study. A multivariate logistic regression analysis of baseline data identified adjusted associations with self-reported property crime soon after release.

**Results:**

Interviews were conducted a median of 23 days post-release with 141 participants. Twenty-eight percent reported property crime in this period and 85 % had injected drugs since release. Twenty-three percent reported injecting at least daily. Reporting daily injecting (adjusted odds ratio (aOR) 4.36; 95 % confidence interval (CI) = 1.45–13.07), illicit benzodiazepine use (aOR = 2.59; 95 % CI = 1.02–5.67), being arrested (aOR = 6.12; 95 % CI = 1.83–20.45) and contact with mental health services (aOR = 4.27; 95 % CI = 1.45–12.60) since release were associated with property crime.

**Conclusion:**

Criminal activity soon after release was common in this sample of PWID, underscoring the need for improved pre-release, transitional and post-release drug use dependence and prevention programmes. Addressing co-occurring mental disorder and poly-pharmaceutical misuse among those with a history of IDU in prison, and during the transition to the community, may reduce property crime in this group.

## Background

Approximately 5600 adults are released from incarceration in the Australian state of Victoria each year [1]. Despite approximately 50 % of Victorian prisoners having a previous incarceration history [1], there is a limited understanding of the factors associated with reoffending following prison release, hampering the development of programmes to reduce crime and re-incarceration. Re-incarceration is a particular issue for people with a history of injecting drug use (IDU). Around half of Australian prisoners report a lifetime history of IDU [2], and studies internationally have identified IDU as a strong predictor of recidivism and re-incarceration among ex-prisoners [3–6]. Further, community-recruited samples of people who inject drugs (PWID) in Australia report frequent engagement in crime, most commonly property crime and drug dealing, alongside significant incarceration histories [7, 8].

The association between drug use and criminal behaviours has been attributed to environmental and social factors, economic motivations and pharmacological/desired drug effects [9–11]. Heroin and benzodiazepines have been reported as the most commonly used drug types among those arrested for property crime in Australia [12]. Among Australian police detainees, 45 % of those attributing crime to heroin use cite economic need as driving criminal behaviour, whereas 74 % of those attributing crime to benzodiazepine use cite disinhibition and intoxication as the reason for their offending [13].

Despite IDU being a strong predictor of recidivism and re-incarceration, there is a paucity of research specifically examining drug use and reoffending among people released from prison with a history of IDU. This gap in knowledge impedes the development of evidence-based programmes to prevent ongoing criminal behaviour in this high-risk group. Given that property crime makes up the largest proportion of receptions into prison in Victoria [1], we aimed to explore the correlates of self-reported property crime in the weeks immediately following prison release in a cohort of ex-prisoners with a history of IDU.

## Methods

This paper presents baseline data from a prospective cohort of recently released prisoners with a history of IDU in the state of Victoria, Australia.

### Participants

Recruitment and baseline data collection occurred between February and November 2009. Eligibility criteria included recent (past 4 weeks) release from prison with a minimum incarceration period of 1 month, at least monthly drug injection in the 6 months prior to incarceration, and residing in metropolitan Melbourne at the time of recruitment. Participants were recruited via (1) targeted field recruitment from street-based drug markets, (2) direct referral from community service workers and (3) snowball sampling. Interviews were conducted at fixed site service providers or mutually convenient locations (e.g. cafes). Trained field researchers screened participants for eligibility, and written informed consent was obtained prior to survey administration. Data were collected via handheld personal digital assistants programmed with Questionnaire Development System Version 2.6.1 software (Nova Research Company, MD, USA), and interviews took a median of 40 min to complete. Participants were reimbursed AU$30 for their time and travel expenses according to standard research practice in Australia [14, 15].

The study was approved by the Victorian Department of Human Services Human Research Ethics Committee and the Victorian DOJ Human Research Ethics Committee.

### Measures

A structured, researcher-administered questionnaire was used to elicit information on socio-demographic characteristics, pre- and post-release utilisation of health and social support services, pre- and post-release use of alcohol and other drugs, involvement in risk behaviours (e.g. injecting, crime) and various health and welfare indicators. Psychological distress was assessed using the Kessler Psychological Distress Scale (K10) [16]. Survey questions were informed by the experience of the research team and include questions commonly asked in studies of PWID and routine surveillance conducted with similar populations [7, 14]. Questionnaires were piloted with the target population and refined before study implementation.

### Analysis

Descriptive analyses of socio-demographics, drug use, criminogenic outcomes and health indicators were undertaken for the whole sample and disaggregated by reported engagement in any property crime since release. Associations between socio-demographic characteristics, health indicators, post-release drug use, police contact and other types of crime with property crime were assessed through univariable logistic regression.

Variables were selected for entry into a multivariable logistic regression model based on strength of univariable association with property crime and/or evidence from previous research. We used backward elimination to identify factors independently associated with property crime, controlling for gender. Variables significant at *p* < 0.05 were retained in the final model. A test for co-linearity (variance inflation factor) was used to eliminate co-linear variables from the model prior to backward elimination. Analyses were conducted using Stata Version 11.1 (StataCorp LP, TX, USA).

## Results

One hundred and forty-one ex-prisoners were recruited to the study, and baseline interviews occurred a median of 23 days (interquartile range [IQR] 15–33) following prison release. Most participants were male (81 %) with a median age of 30 years (range 19–55) (Table 1). The sample was characterised by educational disadvantage, unstable accommodation, mental health issues and extensive incarceration histories. Over one quarter (28 %) of participants reported engagement in property crime since release (Table 1).

**Table 1:** Unadjusted correlates of property crime—socio-demographics and health indicators

Variable	Total sample	Not reported property crime	Reported property crime	Unadjusted	
	*N* = 141 (%)	*n* = 101 (%)	*n* = 40 (%)	OR	95 % CI
Male	114 (81)	83 (82)	31 (78)	1.34	0.54–3.29
Aged ≥30 years	76 (54)	55 (54)	21 (52)	0.92	0.44–1.92
Indigenous	7 (5)	5 (5)	2 (5)	1.01	0.19–5.43
Completed year ≤9 education	50 (35)	32 (32)	18 (45)	1.76	0.83–3.73
Unstable accommodation^a^	51 (36)	32 (31)	19 (47)	1.95	0.92–4.13
Health indicators					
Drug overdose since release	13 (9)	7 (7)	6 (15)	2.37	0.74–7.55
Very high psychological distress^b^	41 (29)	23 (23)	18 (45)	2.47	1.22–5.81
Current contact with mental health service	26 (18)	12 (12)	14 (35)	3.99	1.64–9.69
Currently prescribed ≥ mental health medication	52 (37)	35 (35)	17 (43)	1.39	0.66–2.95
Visited general practitioner since release	90 (64)	57 (56)	33 (83)	3.64	1.47–9.00
Current opioid substitution therapy	128 (91)	91 (90)	37 (93)	1.35	0.35–5.20

^a^For example, boarding house, motel and staying with friends

^b^Measured from K10 [14]

Participants reporting property crime were generally socio-demographically comparable to those not reporting property crime; however, higher proportions of those reporting property crime reported low educational attainment, unstable accommodation, higher rates of non-fatal overdose, very high psychological distress and contact with mental health services (Table 1).

The majority (85 %) of participants had injected drugs since release; 23 % reported injecting at least daily (Table 2). All participants reporting property crime reported post-release IDU. Heroin was the most commonly used illicit drug, followed by cannabis, benzodiazepines and any form of methamphetamine. Use of all types of illicit drugs was more common among participants reporting property crime, with the exception of crystal methamphetamine. For the purposes of this study, illicit use of pharmaceuticals was defined as obtained from sources other than via one’s own prescription, or used outside the bounds of one’s own prescription. Contact with police, arrest and drug dealing/trafficking were also more common among those reporting property crime (Table 2).

**Table 2:** Unadjusted correlates of property crime since release—drug use and criminal justice indicators

Variable	Total sample	Not reported property crime	Reported property crime	Unadjusted	
	*N* = 141 (%)	*n* = 101 (%)	*n* = 40 (%)	OR	95 % CI
Daily injecting	33 (23)	17 (17)	16 (40)	3.29	1.45–7.48
Used heroin	105 (74)	69 (68)	36 (90)	4.17	1.37–12.73
Used cannabis	89 (63)	59 (58)	30 (75)	2.14	0.94–4.83
Used illicit benzodiazepines	56 (40)	32 (32)	24 (60)	3.23	1.51–6.91
Used methamphetamine (powder/speed)	49 (35)	29 (29)	20 (50)	2.48	1.17–5.28
Used pharmaceutical opioids^a^	21 (15)	12 (12)	9 (23)	2.15	0.83–5.60
Used methamphetamine (crystal/ice)	17 (12)	13 (13)	4 (10)	0.75	0.23–2.46
Spent ≥$100 on drugs/week	82 (58)	48 (48)	34 (85)	6.26	2.42–16.20
Sold drugs	31 (22)	16 (16)	15 (38)	3.19	1.38–7.37
Contact with police	59 (42)	34 (34)	25 (63)	3.28	1.53–7.03
Arrested	20 (14)	6 (6)	14 (35)	8.53	2.98–24.37
Incarcerated ≥3 times	90 (64)	60 (59)	30 (75)	2.60	0.91–7.45

^a^Excludes methadone and buprenorphine, includes licit and illicit use

In multivariable analyses, participants reporting property crime were more likely to report daily injecting, illicit benzodiazepine use, arrest and contact with mental health services (Table 3).

**Table 3:** Adjusted correlates of property crime since release from prison

Variable	Adjusted OR	95 % CI
Male	0.61	0.19–2.01
Daily injecting	4.36	1.45–13.07
Used illicit benzodiazepines	2.59	1.02–6.57
Arrested	6.12	1.83–20.45
Contact with mental health services	4.27	1.45–12.60

## Discussion

This study characterised a cohort of people with a history of IDU recently released from prison and examined the correlates of property crime in this group. Despite the relatively short period of time between prison release and interview, more than one quarter of participants reported property crime since release, with almost all types of illicit drugs being more commonly used by these individuals. These data are consistent with the very high levels of re-incarceration found in another Australian study, where 84 % of a cohort of incarcerated male heroin users were re-incarcerated within 2 years of release (almost twice the rate of the general prison population) [6]. Our findings characterise a specific sub-group of ex-prisoners with a history of IDU engaging in property crime: those who are injecting heroin with high frequency, those who are accessing mental health services (presumably reflecting poor mental health) and those illicitly using benzodiazepines and other drugs. The correlates of crime in this study and the close temporal proximity of property crime to prison release emphasise the need for transitional programmes that effectively address prisoners’ complex health and social issues to address reoffending risk and return to poly-drug use among those with IDU histories.

Our sample demonstrated a pattern of problematic poly-drug use common among samples of PWID. Two specific patterns of substance use were associated with property crime in multivariable analyses: daily injecting and illicit benzodiazepine use. As found by others [13, 17], property crime among these participants is likely to be driven by the need to finance their drug use, particularly among those injecting daily or more often. To the best of our knowledge, this is the first study to identify an association between illicit benzodiazepine use and property crime among people recently released from prison. This finding is consistent with Australian data showing a greater likelihood of illegally sourced income and arrest or imprisonment in the previous year among detainees reporting illicit benzodiazepine use [18]. High prevalence benzodiazepine use reported among PWID in Australia, Europe and the USA [7, 19–23] has given rise to increasing concerns about the adverse consequences of co-occurring opioid and benzodiazepine use [24]. The financial pressure of funding drug purchases combined with benzodiazepine-driven disinhibited criminal behaviours reported by PWID [25] suggests a need for targeted actions for crime prevention. Pre- and post-release programmes for those with substance dependence histories should focus on interventions to reduce poly-drug use alongside cautious pharmaceutical prescribing practices. Recent Australian research showed a majority of PWID reporting non-prescription initiation of benzodiazepines, with medical practitioners as their usual current source of benzodiazepines [26]. The purported ‘over-prescribing’ of benzodiazepines in Australia, their potential diversion within populations of PWID and the associated health risks (e.g. overdose) have prompted calls for more cautious benzodiazepine prescribing and improved prescription monitoring [25, 27, 28]. The association with criminal activity should also factor into such considerations.

Individuals reporting contact with mental health services since release were more likely to report property crime. Mental illness is common among Australian prison populations [29] and often occurs in conjunction with substance use disorders [30]. Previous research indicates that about one quarter of Victorian prisoners had contact with mental health services prior to imprisonment, and males with mental health and substance dependence dual diagnoses were 12 times more likely to be convicted than males in the general population [31, 32]. Recent Australian research also identified the under-ascertainment of mental illness at prison reception, which was influenced by the availability of prison mental health resources [33]. The authors highlighted incarceration as a critical juncture for providing early opportunities to identify mental illness and initiate treatment. Our findings also highlight opportunities to prevent recidivism by targeting individuals with mental health needs through mental health services both before and, crucially, after release from prison.

## Limitations

Our multivariable analyses were limited by the small sample size and thus the limited number of variables that could be retained in the model. However, the range of significant correlates identified using backward elimination provides new insights into the factors associated with crime for this population in the post-release period. Although our sample displayed similar characteristics to other samples of Australian PWID [7, 14], the recruitment of PWID after their release in and around services frequented by PWID means that the sample may not be representative of all people leaving prison with a history of IDU. Results are likely to reflect those at greatest risk of returning to IDU and potentially those at most risk of reoffending and re-incarceration and at greater risk of arrest given the concentration of police activity in these areas. However, in light of very high rates of recidivism and re-incarceration among PWID [6], our findings are highly relevant to informing interventions for a significant proportion of PWID released from prison. Finally, while our data offer insights regarding the potential role of substance use patterns in driving crime in this population, conclusions are limited by the lack of data regarding direct motives for engaging in property crime. Rates of property crime may have been under-reported due to fear of reprisals for disclosing property crime to researchers, despite participants being briefed as to the low risk of this occurring.

## Conclusion

This is the first study to examine the factors associated with post-release property crime among people with a history of IDU in Australia. Our findings demonstrate an association between particular types of reoffending and certain patterns of drug use, arrest and contact with mental health services in the immediate post-release period. The potential role of illicit benzodiazepine use in increasing the risk of engaging in property crime requires further exploration. Further, an improved understanding of the reasons PWID engage in poly-pharmaceutical use (e.g. desire for stronger sedation) may help inform more effective in-prison and community prevention programmes to reduce reoffending and re-incarceration among those with a history of IDU. The high proportion of participants returning to drug use and the association of early post-release property crime with high-frequency injecting, poly-drug use and mental health service access underscore the importance of effective pre- and post-release programmes for offenders with complex health needs. Programmes that focus on rehabilitation across a range of health and welfare domains, particularly those associated with problematic patterns of drug use and mental health, have the potential to reduce rates of reoffending and re-incarceration among this population.

## References

[1] Department of Justice (2009). Statistical profile of the Victorian prison system.

[2] Australian Institute of Health and Welfare (2013). The health of Australia’s prisoners 2012.

[3] Dowden C, Brown S (2002). The role of substance abuse factors in predicting recidivism: a meta-analysis. Psychol, Crime and Law.

[4] Passey M (2003). Evaluation of the Lismore MERIT Pilot Program - final report.

[5] Kinner SA (2006). The post-release experience of prisoners in Queensland. Trends and Issues in Crime and Crim Justice.

[6] Larney S, Toson B, Burns L, Dolan K (2011). Opioid substitution treatment in prison and post-release: effects on criminal recidivism and mortality.

[7] Stafford J, Burns L, Trends AD (2011). Findings from the Illicit Drug Reporting System (IDRS), in Australian Drug Trends Series 2012.

[8] Kinner SA, George J, Campbell G, Degenhardt L (2009). Crime, drugs and distress: patterns of drug use and harm among criminally involved injecting drug users in Australia. Aust N Z J Public Health.

[9] Bennett T, Holloway K, Farrington D (2008). The statistical association between drug misuse and crime: a meta-analysis. Aggression and Violent Behav.

[10] Gottfredson DC, Kearley BW, Bushway SD (2008). Substance use, drug treatment and crime: an examination of intra-individual variation in a drug court population. J Drug Issues.

[11] Goldstein PJ (1985). The drugs/violence nexus: a tripartite conceptual framework. J Drug Issues.

[12] Gaffney A, Jones W, Sweeney J, Payne J (2010). Drug use monitoring in Australia: 2008 annual report on drug use among police detainees.

[13] Payne J, Gaffney A (2012). How much crime is drug and alcohol related? Self-reported attributions of police detainees. Trends and Issues in Crime and Crim Justice.

[14] Horyniak D, Higgs P, Jenkinson R, Degenhardt L, Stoové M, Kerr T, Hickman M, Aitken C, Dietze P (2012). Establishing the Melbourne Injecting Drug User Cohort Study (MIX): rationale, methods, and baseline and twelve-month follow-up results. Harm Reduction J.

[15] Fry C, Ritter A, Baldwin S, Bowen K, Gardiner P, Holt T, Jenkinson R, Johnston J (2005). Paying research participants: a study of current practices in Australia. J Med Ethics.

[16] Kessler RC, Barker PR, Colpe LJ, Epstein JF, Gfroerer JC, Hiripi E, Howes MJ, Normand S-LT, Manderscheid RW, Walters EE, Zaslavsky AM (2003). Screening for serious mental illness in the general population. Arch Gen Psychiatry.

[17] Nielsen S, Bruno R, Carruthers S, Fishcer J, Lintzeris N, Stoové M (2008). Investigation of pharmaceutical misuse amongst drug treatment clients.

[18] Loxley W. Benzodiazepine use and harms among police detainees in Australia. Trends and Issues in Crime and Crim Justice. 2007;336. Available at: http://aic.gov.au/publications/current%20series/tandi/321-340/tandi336.html.

[19] Chen KW, Berger CC, Forde DP, D’Adamo C, Weintraub E, Gandhi D (2011). Benzodiazepine use and misuse among patients in a methadone program. BMC Psychiatry.

[20] Eiroa-Orosa FJ, Haasen C, Verthein U, Dilg C, Schäfer I, Reimer J (2010). Benzodiazepine use among patients in heroin-assisted vs. methadone maintenance treatment: findings of the German randomized controlled trial. Drug Alcohol Depend.

[21] Fernandez Sobrino AM, Fernández Rodríguez V (2009). Benzodiazepine use in a sample of patients on a treatment program with opiate derivatives (PTDO). Adicciones.

[22] Lavie E, Fatséas M, Denis C, Auriacombe M (2009). Benzodiazepine use among opiate-dependent subjects in buprenorphine maintenance treatment: correlates of use, abuse and dependence. Drug Alcohol Depend.

[23] Specka M, Bonnet U, Heilmann M, Schifano F, Scherbaum N (2011). Longitudinal patterns of benzodiazepine consumption in a German cohort of methadone maintenance treatment patients. Human Psychopharmacol: Clin Exp.

[24] Jones JD, Mogali S, Comer SD (2012). Polydrug abuse: a review of opioid and benzodiazepine combination use. Drug Alcohol Depend.

[25] Smith B, Miller P, O’Keefe B, Fry C. Benzodiazepine and pharmaceutical opioid misuse and their relationship to crime: Victorian report. NDLERF Monograph 23, 2007, National Drug Law Enforcement Research Fund: Canberra. Available at: http://www.ndlerf.gov.au/publications/monographs/monograph-23.

[26] Nielsen S, Bruno R, Degenhardt L, Stoové M, Fischer J, Carruthers S, Lintzeris N (2013). The source of pharmaceuticals for problematic users of prescription opioids and benzodiazepines. Med J Aust.

[27] Nielsen S, Dietze P, Lee N, Dunlop A, Taylor D (2007). Concurrent buprenorphine and benzodiazepines use and self-reported opioid toxicity in opioid substitution treatment. Addiction.

[28] Shand F, Campbell G, Hall W, Lintzeris N, Cohen M, Degenhardt L (2013). Real time monitoring of Schedule 8 medicines in Australia. Med J Aust.

[29] White P, Whiteford H (2006). Prisons: mental health institutions of the 21st century?. Med J Aust.

[30] Butler T, Indig D, Allnutt S, Mamoon H (2011). Co-occurring mental illness and substance use disorder among Australian prisoners. Drug Alcohol Rev.

[31] Mullen PE, Burgess P, Wallace C (2000). Community care and criminal offending in schizophrenia. Lancet.

[32] Mullen P (2001). A review of the relationship between mental disorders and offending behaviours on the management of mentally abnormal offenders in the health and criminal justice system.

[33] Schildersab MR, Ogloff J (2014). Review of point-of-reception mental health screening outcomes in an Australian prison. J Forensic Psychiatry Psychol.

